# Prevalence and risk factors for lifetime suicide ideation, plan and attempt in Chinese men who have sex with men

**DOI:** 10.1186/s12888-016-0830-9

**Published:** 2016-04-29

**Authors:** Huijuan Mu, Yanxia Li, Li Liu, Jun Na, Liya Yu, Xuejuan Bi, Xiaoxia An, Yuan Gu, Yan Zhou, Shuang Li, Rui Zhang, Chao Jiang, Guowei Pan

**Affiliations:** Institute of Chronic Disease, Liaoning Provincial Center for Disease Control and Prevention, Sayang Road 242, Heping District, Shenyang, 110005 P.R. China; Anshan Municipal Center for Disease Control and Prevention, Anshan, P.R. China; Benxi Municipal Center for Disease Control and Prevention, Benxi, P.R. China; Shenyang Municipal Center for Disease Control and Prevention, Shenyang, P.R. China; Dandong Municipal Center for Disease Control and Prevention, Dandong, P.R. China; Department of Psychiatry, Dalian Medical University, Dalian, P.R. China

**Keywords:** Men who have sex with men, Suicidal behavior, Psychiatric disorder, Comorbidity

## Abstract

**Background:**

To describe the level and risk factors for suicidal behaviors in Chinese men who have sex with men (MSM).

**Methods:**

A total of 807 MSM were recruited using a respondent-driven sampling method from Anshan, Benxi, Dandong, and Shenyang cities in northeastern China.

**Results:**

Chinese MSM had lifetime prevalences of suicide ideation (18.3 %), plan (8.7 %) and attempt (4.6 %) that were about 2.8, 5.8 and 5.8 times greater than that of male adults in the general population of China. The MSM with any psychiatric disorders were 4–7 times more likely to think about, plan or attempt suicide than those MSM with no disorder, and there was a clear relationship between the number of comorbid disorders and suicidal behaviors. Multiple regression analysis showed that major depression, bipolar disorder, dysthymia and alcohol use disorder significantly increased the risk for suicide ideation, but not for suicide attempt. Drug dependence disorder, panic disorder and generalized anxiety disorder significantly increased the risk for suicide attempt, but not for suicide ideation. More advanced education reduced the risk of suicidal behaviors, sexual orientations revealed to or discovered by family members or friends significantly increased risk of these suicidal behaviors.

**Conclusions:**

Chinese MSM have significantly increased risk for suicidal behaviors, mental disorders and their comorbidities could be the largest risk factors for the elevated suicidal behaviors in Chinese MSM. Reducing the family and social stigma and rejection of homosexual behavior and early detection and effective treatment of psychiatric disorders and their comorbidities in MSM may help to decrease suicidal behaviors of Chinese MSM.

## Background

Studies of men that had same-sex sexual identities, behaviors (men who have sex with men, MSM), and orientations indicated that men have greater risk for suicidal behaviors when they have homosexual partners [1–5]. In agreement, a 12-year prospective cohort study in Denmark reported a nearly 8-fold higher risk for suicide mortality of men in same-sex registered domestic partnerships than in men with histories of heterosexual marriage [6].

The presence of a psychiatric disorder is the strongest and most consistent risk factor for suicidal behavior in many countries [7]. Previous research reported that MSM are vulnerable to mental disorders, including anxiety, depression, and substance misuse disorders, because they experience high levels of social stigma and prejudice [1, 2, 8, 9], anti-gay harassment, discrimination, and victimization [10, 11]. It may be that external factors, such as stigmatization and discrimination, directly lead to psychiatric disorders and suicidality; alternatively, these external factors might have indirect effects via internal factors (like internalized homophobia), as indicated by theories linking minority-specific stress to negative physical and mental health outcomes [12].

Previous studies reported different associations between individual mental disorders and suicidal behaviors. For example, depression is associated with suicide ideation, but anxiety, conduct disorder, and substance use disorders are associated with suicide plan and suicide attempt [13]. Mental disorders are highly comorbid [14], and MSM have greater risks of comorbidities than heterosexual men [15]. Some general population studies reported strong dose–response relationships between the number of mental disorders and the risk of suicidal behaviors; subjects with multiple disorders had odds ratios (OR) for attempted suicide that was several times higher than that of subjects with a single disorder [13, 16]. However, few studies have assessed the differences in the effects of individual and multiple psychiatric disorders on suicidal behaviors in MSM.

Suicide is the fifth leading cause of death in China, with an annual mortality of 23 per 100,000; the 287,000 annual deaths from suicide in China account for about one-fifth of all suicides world-wide [17, 18]. The tolerance to homosexual behavior in general Chinese people is still low. Two surveys showed that 57.2 % doctors and over 82.0 % college students believed that homosexual behaviors were a psychiatric disease, 37.0 % doctors and 11.2 % students would break off the friendship when knowing their friends’ homosexuality [19, 20]. Homosexuality has not been removed from Chinese classification and diagnostic criteria of mental disorders until 2001 [21]. Although 84.7 % Chinese MSM want to have same sex marriage, this issue has been discussed only in the academic society [22].

There are an estimated 17.8 million MSM in China in 2002 [23], and recent studies, which used different definitions of suicidal behaviors, reported increased prevalence of suicide ideation, suicide plan, or suicide attempt in Chinese gays or bisexuals [24–29]. However, these results cannot be compared with those of the general Chinese population or with MSM in other countries because these previous studies did not use a standardized instrument for assessment of suicidal behaviors, such as the World Mental Health Composite International Diagnostic Interview (WMH-CIDI) [30]. Despite the growing interest in identification of risk factors for suicidality in the general population of China, few studies have systematically assessed the risk factors for suicidal behaviors, such as psychiatric disorders, gay-related stressful events, and sexual orientation, in MSM from China. Therefore, there is an incomplete understanding of the prevalence and risk factors for suicidal behaviors in Chinese MSM.

This study was designed to describe the prevalence of lifetime suicide ideation, plan and attempt in 807 MSM from four cities in northeastern China, and to assess the following hypotheses: *(i)* Chinese MSM have significantly higher risk of suicidal behaviors than the general male population; *(ii)* some social and demographic factors, such as disclosed or identified homosexual identity, increase the risk for suicidal behaviors; *(iii)* psychiatric disorders are significantly related to the risks of suicide behaviors, and there are significant differences in the relationship of individual psychiatric disorders with different suicidal behaviors.

## Methods

The locations of the study areas and selection of MSM were described previously [15]. In brief, 807 MSM who lived or worked in four cities of Liaoning province in northeastern China (Anshan, Benxi, Dandong, and Shenyang) were recruited using a standardized respondent-driven sampling (RDS) procedure from April 2008 to January 2009. Respondents were included if they had oral or anal sexual relations with another man during the previous 12 months, were 18 to 65 years-old, agreed to complete a questionnaire, and provided blood samples for testing. After complete description of the study to each respondent, written informed consent was obtained. Each subject was interviewed personally by trained interviewers. Age, socio-demographic characteristics, sexual identity, and disclosure of homosexual identity were recorded using a structured questionnaire. Names and other personal information, including identification numbers, were not recorded.

### Definition of variables

The following socio-demographic factors were recorded: age, education (<10, 10–12, ≥13 years), sexual identity (homosexual, bisexual, heterosexual, unconfirmed), marital status (single, married, cohabitation with a male partner, divorced), disclosure of homosexual identity (yes/no), and homosexual behavior known by parents or wife (yes/no).

### Psychiatric disorders

We used a Chinese version of the Composite International Diagnostic Interview Version 1.0 (CIDI 1.0) [31] in the personal interviews to document psychiatric disorders. This test is a fully structured diagnostic interview designed for use by trained interviewers who are not clinicians, and generates diagnoses based on the ICD-10 and DSM-IIIR diagnostic systems. Core disorders included mood disorders (major depression, dysthymia, and bipolar disorder), anxiety disorders (panic disorder, generalized anxiety disorder (GAD], simple phobia, social phobia, agoraphobia, and obsessive compulsive disorder (OCD), and substance use disorders (alcohol and drug abuse and dependence). Diagnoses were made without diagnostic hierarchy rules [32], so that individuals could meet the criteria for any single disorder regardless of the presence of other disorders.

### Suicidal behaviors

Suicidality is assessed in a section of the World Mental Health CIDI (WMH-CIDI) by a series of questions about suicidal behaviors including suicide ideation, plan and attempt [30]. Informants were asked specific questions to assess suicide ideation (“Have you ever seriously thought about killing yourself and, if so, have you had these thoughts in the past 12 months?”), suicide plan (“Have you ever made a plan for committing suicide and, if so, have you made such a plan in the past 12 months?”), and suicide attempt (“Have you ever attempted suicide and, if so, have you attempted suicide in the past 12 months?”). Respondents who reported attempting suicide in the previous 12 months were asked to describe the seriousness of the action by indicating which of the following 3 statements best described this event: “I made a serious attempt to kill myself and it was only luck that I did not succeed”; “I tried to kill myself, but knew the method was not fool-proof”; and “My attempt was a cry for help. I did not intend to die”. Respondents who indicated either of the first 2 statements were considered to have made suicide attempts; respondents who indicated the third statement were considered to have made suicide gestures [30].

### Statistical methods

Demographic variables and psychiatric disorders were recorded in all subjects to assess their effect on the lifetime risk of suicide ideation, plan and attempt. Co-morbidity was defined as the presence of more than one psychiatric disorder. Because of the small difference between RDS weighted rates and crude rate [15], we calculate crude prevalence in the results. First, we conducted bivariate logistic regression analyses of the 3 lifetime suicidal behaviors for calculation of crude odds ratios (ORs) and 95 % confidence intervals (CI) to examine their associations with each of the sociodemographic factors and lifetime psychiatric disorders with adjustment for age. Second, we conducted stepwise logistic regression including all the sociodemographic factors and psychiatric disorders to calculate the adjusted ORs. All analyses were performed using SPSS, and all 2-sided significance tests were evaluated at the 0.05 level.

## Results

Table 1 shows that 71.3 % of MSM were under 30 years of age, 13.4 % were married, 11.4 % lived with male partners, 47.2 % were gay, 40.3 % were bisexual, and 10.9 % had unconfirmed sexual identities. In addition, 17.4 % reported that their families knew about their homosexual behavior, 22.9 % were open about their homosexual behavior, 35.2 % had diagnosed mental disorders, 15.4 % had 2 or more comorbid disorders. The lifetime prevalence rates of suicide ideation, plan, and attempt were 18.3 %, 8.7 % and 4.6 %, respectively.

**Table 1:** Demographic characteristics and lifetime prevalence of suicide ideation, suicide plan, and suicide attempt of 807 Chinese men who have sex with men

Characteristic	N	%
Age, yrs.		
18–29	575	71.3
30–39	133	16.5
40–64	99	12.3
Education, yrs.		
<10	255	31.6
10–12	322	39.9
≥13	230	28.5
Relationship status		
Single	571	70.8
Married	108	13.4
Cohabitation	92	11.4
Divorced	36	4.5
Sexual identity		
Homosexual	381	47.2
Heterosexual	13	1.6
Bisexual	325	40.3
Unconfirmed	88	10.9
Homosexual behavior known by family members	140	17.4
Disclosure of homosexual identity	185	22.9
Lifetime psychiatric disorders		
Mood disorder	92	11.4
Major depression	55	6.8
Dysthymia	28	3.5
Bipolar disorder	19	2.4
Anxiety disorder	169	20.9
Panic disorder	14	1.7
GAD	6	0.7
Simple phobia	69	8.6
Social phobia	41	5.1
Agoraphobia	93	11.5
Alcohol disorder	167	20.7
Alcohol abuse	43	5.3
Alcohol dependence	124	15.4
Drug disorder	26	3.2
Drug dependence	22	2.7
Drug abuse	5	0.6
Any disorder	284	35.2
Comorbidities		
1	160	19.8
2	61	7.6
3	28	3.5
≥4	35	4.3
Lifetime suicide behaviors		
Ideation	148	18.3
Plan	70	8.7
Attempt	37	4.6

Table 2 shows the ORs for lifetime suicide ideation, plan and attempt in MSM according to different sociodemographic characteristics and lifetime psychiatric disorders. The prevalence of suicide plan was significantly greater in subjects who were 40 to 64 years-old (OR = 2.9, 95 % CI = 1.1-7.5) and significantly less in those who had 13 or more years of education (OR = 0.5, 95CI = 0.3-1.0). Heterosexual marriage and divorce were unrelated to suicidal behaviors. However, cohabitation with a same sex partner was positively associated with all 3 suicidal behaviors (ideation: OR = 2.1, 95 % CI = 1.3-3.4; plan: OR = 2.1, 95 % CI =1.1-4.0; attempt: OR = 2.3, 95 % CI = 1.0-5.0) and disclosure of homosexual behavior was also positively associated with all 3 suicidal behaviors (ideation: OR = 2.1, 95 % CI = 1.5-3.2; plan: OR = 2.8, 95 % CI = 1.7-4.7; attempt: OR = 2.4, 95 % CI = 1.2-4.7). Subjects whose homosexual behavior was known to family members had increased risk of suicide ideation (OR = 1.7, 95 % CI = 1.1-2.8) and suicide plan (OR = 2.4, 95 % CI = 1.4-4.2), but not suicide attempt.

**Table 2:** Risk factors for lifetime suicide ideation, plan and attempt of 807 Chinese men who have sex with men

Risk Factor	Ideation			Plan			Attempt		
	OR	95 %	CI	OR	95 %	CI	OR	95 %	CI
Age, yrs.									
18–29	1.0			1.0			1.0		
30–39	1.1	0.6	2.0	1.3	0.5	3.0	2.6	0.6	11.0
40–64	1.5	0.8	3.0	2.9*	1.1	7.5	2.3	0.5	11.6
Education, yrs.									
<10	1.0			1.0			1.0		
10–12	0.9	0.6	1.4	0.7	0.4	1.2	0.7	0.4	1.5
≥13	0.6	0.4	1.0	0.5*	0.3	1.0	0.4	0.2	1.0
Orientation									
Heterosexual/unconfirmed	1.0			1.0			1.0		
Homosexual	1.5	0.9	2.7	1.8	0.7	4.4	2.2	0.7	7.4
Bisexual	0.9	0.5	1.7	1.3	0.5	3.3	1.0	0.3	3.8
Relationship status									
Single	1.0			1.0			1.0		
Married	0.6	0.3	1.1	0.7	0.3	1.7	0.2	0.0	1.5
Cohabitation	2.1*	1.3	3.4	2.1*	1.1	4.0	2.3*	1.0	5.0
Divorced	1.7	0.8	3.6	2.0	0.7	5.3	0.6	0.1	4.7
Disclosure of homosexuality	2.1*	1.5	3.2	2.8*	1.7	4.7	2.4*	1.2	4.7
Homosexuality known by family members	1.7*	1.1	2.8	2.4*	1.4	4.2	2.0	0.9	4.2
Psychiatric disorder									
Any mood disorder	12.6*	7.8	20.3	9.5*	5.5	16.3	6.1*	3.1	12.3
Major depression	13.1*	7.1	23.9	10.8*	5.9	19.9	6.0*	2.7	13.1
Dysthymia	7.6*	3.5	16.7	4.6*	2.0	10.9	2.6	0.8	9.1
Bipolar	5.2*	2.1	13.1	2.9	0.9	9.0	4.2*	1.2	15.0
Any anxiety disorder	4.6*	3.1	6.8	5.4*	3.3	9.0	5.4*	2.8	10.7
Panic disorder	8.5*	2.8	25.7	11.6*	3.9	34.1	25.4*	8.4	77.1
GAD	9.1*	1.7	50.3	11.0*	2.2	55.3	22.6*	4.4	115.9
Simple phobia	2.6*	1.6	4.1	2.5*	1.4	4.7	1.5	0.6	3.8
Social phobia	9.2*	4.7	17.8	6.6*	3.3	13.2	7.4*	3.2	17.0
Agoraphobia	2.6*	1.6	4.1	2.5*	1.4	4.7	1.5	0.6	3.8
OCD	27.8*	3.3	232.7	28.3*	5.4	148.6	3.5	0.4	30.2
Alcohol disorder	4.3*	2.9	6.3	4.6*	2.8	7.6	4.0*	2.0	7.7
Alcohol dependence	3.4*	2.2	5.1	4.5*	2.6	7.5	4.1*	2.1	8.2
Alcohol abuse	3.9*	2.1	7.3	2.2	0.9	5.1	1.6	0.5	5.5
Drug disorder	7.8*	3.5	17.7	9.0*	3.9	20.4	11.5*	4.6	28.7
Drug dependence	8.5*	3.5	20.7	10.1*	4.2	24.3	11.7*	4.5	30.9
Drug abuse	6.8*	1.1	41.0	2.7	0.3	24.1	14.6*	2.4	90.3
Any disorder	6.3*	4.3	9.3	6.8*	3.9	12.1	4.1*	2.0	8.3
Comorbidities									
0	1.0			1.0			1.0		
1	3.2*	2.0	5.1	3.1*	1.5	6.3	0.8	0.2	2.9
2	8.1*	4.5	14.7	5.8*	2.5	13.4	5.5*	2.1	14.6
3	16.8*	7.4	38.2	16.5*	6.7	41.2	9.3*	3.0	28.5
≥4	27.2*	12.3	60.3	31.5*	13.9	71.6	17.0	6.7	43.2

*GAD* general anxiety disorder, *OCD* obsessive compulsive disorder

*p < 0.05

All psychiatric disorders and their comorbidity were associated with significantly increased risk of suicide ideation; the ORs were especially high in subjects with OCD (OR = 27.8, 95 % CI = 3.3-232.7) and with 4 or more comorbidities (OR = 27.2, 95 % CI = 12.3-60.3). In addition, except for bipolar disorder, alcohol dependence and drug abuse, all disorders increased the risk of suicide plan; the ORs were especially high for subjects with OCD (OR = 28.3, 95 % CI = 5.4-148.6), with 3 (OR = 16.5, 95 % CI = 6.7-41.2), and with 4 or more comorbidities (OR = 31.5, 95 % CI = 13.9-71.6). The results for suicide attempt were more mixed. There were no significant relationships of suicide attempt with dysthymia, simple phobia, agoraphobia, OCD, alcohol dependence, and 1 comorbidity. All other disorders were significantly associated with suicide attempt; the ORs were especially high in subjects with panic disorder (OR = 25.4, 95 % CI = 8.4-77.1), GAD (OR = 22.6, 95 % CI = 4.4-115.9), and 4 or more comorbidities (OR = 17.0, 95 % CI = 6.7-43.2).

Table 3 shows the results of multiple logistic regression analysis. More years of education provided significant or nearly significant protection against all 3 suicidal behaviors (OR = 0.3 to 0.6). Disclosure of homosexual behavior was positively associated with increased risk of suicide ideation (OR = 2.0, 95 % CI = 1.3-3.2) and suicide plan (OR = 1.9, 95 % CI = 1.0-3.5). When family members knew about a subject’s homosexual behavior, this was associated with significantly increased risk of suicide plan (OR = 3.0, 95 % CI = 1.6-5.6) and suicide attempt (OR = 2.4, 95 % CI = 1.0-5.7). Major depression was associated with significantly increased the risk of suicide ideation (OR = 8.2, 95 % CI = 4.2-16.2) and suicide plan (OR = 7.2, 95 % CI = 3.6-14.5), but not for suicide attempt. Bipolar disorder (OR = 4.8, 95 % CI = 1.8-13.0) and dysthymia (OR = 3.8, 95 % CI = 1.5-9.5) was associated with significantly increased risk of suicide ideation, but not for suicide plan and attempt.

**Table 3:** Multiple logistic regression analysis of all disorders and comorbidities for lifetime suicide behaviors of 807 Chinese men who have sex with men

	Ideation			Plan			Attempt		
	OR	95 %	CI	OR	95 %	CI	OR	95 %	CI
Major depression	8.2*	4.2	16.2	7.2*	3.6	14.5	2.0	0.6	6.2
Bipolar	4.8*	1.8	13.0	1.9	0.5	6.8	2.0	0.4	10.0
Dysthymia	3.8*	1.5	9.5	0.7	0.2	2.4	0.3	0.0	1.7
Social phobia	3.3*	1.5	7.5	1.2	0.4	3.4	4.3*	1.5	12.0
Panic disorder	2.9	0.8	11.2	6.2*	1.7	21.9	25.0*	7.4	84.3
GAD	4.5	0.5	42.8	6.4*	0.9	44.4	19.6*	3.0	128.0
OCD	6.77	0.5	89.6	16.6*	2.6	104.8	0.2	0.0	7.9
Alcohol dependence	2.0*	1.2	3.4	2.7*	1.5	5.2	1.2	0.4	3.2
Alcohol abuse	4.5*	2.2	9.1	2.9*	1.1	7.6	1.6	0.4	6.7
Drug dependence	2.2	0.7	7.0	2.8	0.9	8.9	11.3*	3.7	34.3
Education years≧13 yr	0.6*	0.3	0.9	0.5*	0.2	1.0	0.3	0.1	1.0
Disclosure of homosexuality	2.0*	1.3	3.2	1.9	1.0	3.5	1.8	0.8	4.4
Homosexuality known by others	1.7	1.0	2.9	3.0*	1.6	5.6	2.4*	1.0	5.7

Variables with asterisks were selected in the logistic stepwise regression model, and these variables were adjusted for each other in the same model. The other variables (without asterisks) were forced into in the final model and adjusted for each other and for age.

*GAD* general anxiety disorder, *OCD* obsessive compulsive disorder

*p < 0.05

Panic disorder and GAD was associated with significantly increased risk for suicide plan and attempt, but not for suicide ideation. Social phobia significantly had significantly increased risk for suicide ideation and attempt, but not for suicide plan. OCD was significantly associated with increased risk for suicide plan (OR = 16.6, 95 % CI = 2.6-104.8). Alcohol dependence and abuse was associated with significantly increased risk for suicide ideation and plan, but not for suicide attempt. Drug dependence was associated with significantly increased risk for suicide attempt (OR = 11.3, 95 % CI = 3.7-34.3), but not for suicide ideation and plan.

## Discussion

The highest prevalence of lifetime suicide ideation, plan and attempt from five surveys that used the same CIDI questionnaire and the same definitions of suicidal behaviors in Chinese male adults were 6.4 %, 1.5 % and 0.8 %, respectively [33–37]. Therefore, we can roughly estimate that Chinese MSM (18.3 %, 8.7 % and 4.6 %, respectively) were about 2.8, 5.8 and 5.8 times more likely to think about, plan, and attempt suicide than the general male population. Compared to the results of Chinese male adults in the World Mental Health (WMH) survey in Beijing and Shanghai (ideation: 2.3 %, plan: 0.9 %, attempt: 0.7 %, [33]) and in Taiwan (ideation: 3.30 %, attempt: 0.39 % [38]), we believe the above estimations about the increased risk of suicidal behaviors in Chinese MSM relative to the general population may be considered as very conservative. This provides further evidence that MSM or sexual minority men (SMM; men who endorse same-sex attraction, same-sex behavior, or a gay identity) have increased risk for suicidality [1–5, 12, 39–41].

The previously reported lifetime prevalences of suicide ideation (3.1 %), plan (0.9 %), and attempt (1.0 %) in Chinese adults were only about 1/3 of the average levels of the 17 countries in the WMH surveys (9.2 %, 3.1 %, and 2.7 % respectively, [12]). Similarly, the lifetime rates of suicide ideation (18.3 %) and suicide attempt (4.6 %) in our cohort were about half of those for gay and bisexual men in North America and Europe (suicide ideation: 40–55 %, suicide attempt: 8–25 %, [42]. Similarly, Latino and Asian American gay and bisexual adults have significantly lower rates of 12-month (2.4 %) and lifetime (8.0 %) suicide attempts than their counterparts in the USA [42, 43]. Thus, although there are large differences in the prevalences of suicidal behaviors in different countries, our results support the view that within a country, the rates of suicidal behaviors in adults who report same-sex behaviors is 3–5 times higher than in those who report no same-sex behaviors [5, 44, 45].

As expected, we found that the MSM with any psychiatric disorders were 4–7 times more likely to think about, plan or attempt suicide than those MSM with no disorder (Table 2). Compared to urban male adults, this cohort had a 2.8, 5.5, 3.0, and 2.4 fold greater risks for any psychiatric disorder, anxiety disorder, mood disorder and alcohol use disorder, respectively [15], and we found that 68–76 % of Chinese MSM with suicidal behaviors had psychiatric disorders. Therefore, we believed that the significantly elevated mental disorders and their comorbidities constitute the single largest risk factor for suicidal behavior in Chinese MSM. To the knowledge of the authors, this is the first study on the associations between lifetime psychiatric disorders and suicidal behaviors among Chinese MSM. We found that 26.02 % of the study participants experienced gay related stressful events (GRSEs) during the previous three months, and they had higher scores depression symptom, anxiety symptom, but lower social support score, compared to the national norms [46]. These results were consistent with the previous findings of a few studies in Chinese MSM [47–49], suggesting the “synthesis” of these social and psychological problems that create these health disparities.

Our bivariate analysis indicated that most mental disorders were associated with significantly increased risk of suicidal behaviors, with large variations in the ORs (Table 2). Interestingly, the multiple regression analysis indicated that the presence of major depression, bipolar disorder, dysthymia and alcohol disorders significantly increased the risk for suicide ideation, but had no significant effects on suicide attempt (Table 3). On the other hand, drug dependence disorders, panic disorder and GAD significantly increased the risk for suicide attempt, but had no significant effects on suicide ideation. These results are similar to those of studies of general populations, in that there are large differences in the relationships between individual psychiatric disorders and suicidal ideation, plan and attempt [13, 16]. Our results support the hypothesis that mood disorder mainly provoke suicide ideation, but that disorders characterized by anxiety and poor impulse control (drug dependence) increase the risk of acting on suicidal thoughts or plans [7, 13, 50–52]. Some family and genetic studies suggested that mental disorders are mainly responsible for the co-occurrence of suicide ideation within a family, but not for the tendency to act on suicidal thoughts. Instead, a distinct genetic component perhaps related to the presence of impulsive-aggressive traits, is associated with suicide attempt [53, 54].

We observed a clear relationship of the number of comorbid disorders with suicidal behaviors (Table 2), and this cohort had a 4.2 fold greater risk of comorbidity than urban male adults [15]. Multiple disorders are associated with more distress, psychological impairment, and disease burden [55, 56], and lead individuals to attempt suicide in an effort to escape intolerable distress regardless of the specific source of that distress. This is consistent with an escape model of suicide [57, 58]. Moreover, it will be more difficult to successfully reduce suicidality in MSM than in the general population because treating people with more comorbidities requires simultaneous treatment of multiple disorders, rather than a single disorder [13, 16].

Consistent with previous findings in general and MSM populations [7, 33, 59], we found that more advanced education reduced the risk of suicidal behaviors in Chinese MSM. Thus, more education and the concomitant better socioeconomic status appeared to improve coping capability. Similar to the situation in Japanese MSM [60], only 22.9 % of the Chinese MSM in our cohort revealed their sexual orientations to friends and family members, and only 17.4 % reported that their homosexual behaviors were known or discovered by family members. This confirms a previous report that most Chinese MSM keep their homosexuality secret and do not seek help or support from family members [61]. Concealment of one’s homosexuality is a common coping strategy in which the individual aims to avoid social stigmatization, but it can lead to increased stress [62]. The significant associations between disclosure/identification of homosexuality and suicidal behaviors indicate that failure to maintain the secret or being identified by others can lead to a “private hell” [63]; this can lead to higher levels of harm, shame, guilt, social ostracization, and loss of support, and thereby increase the risk of suicidal behaviors [60]. We hypothesize that family pressures are particularly severe in China, which has the one–child–one–couple policy; a homosexual lifestyle, with no marriage or children is considered unacceptable in traditional Chinese culture [61].

It is important to note that previous studies which reported significantly elevated suicidality among MSM and SMM used different definitions of these conditions, including same-sex attraction, behavior, orientation, or identity [1–5, 12, 38–41, 64]. In fact, there is limited understanding of which dimension of sexual orientation or homosexual behavior is most meaningfully related to suicidal behavior [1]. A recent study of adolescents found that the 12-month rate of suicidal ideation and attempt were significantly higher in individuals with gay/lesbian/bisexual and unsure identities than those who reported same-sex behavior but heterosexual identity, and heterosexuals who did not engage in same-sex behavior [65]. Only 13 of the subjects in our cohort reported heterosexual orientation. Thus, our observed rates of suicide ideation (2/13), plan (1/13), and attempt (0/13) in these 13 subjects are based on a very small sample. Nonetheless, our results support the view that MSM who have heterosexual identities may have lower risk of suicidal behaviors. Clearly, future studies with more subjects are needed to further examine the association between self-reported sexual orientation and suicidal behaviors.

There are several limitations of this study. First, we did not select a separate control group of local males to calculate age adjusted relative risks (RRs); instead, we roughly estimated the RRs by comparing with the highest corresponding rates of suicidal behaviors from five studies that used the same WMH-CIDI definitions about suicidal behaviors. We could not estimate the possible bias from this practice, although our procedure tend to underestimate the RRs. Second, we used an older version of the CIDI, which was based on DSM-III-R rather than DSM-IV or DSM-V. Because we calculated the ORs of individual psychiatric disorders and comorbidities as risk factors within MSM samples, the application of CIDI 1.0 did not affect the comparability of mental disorders and the validity of the results. Third, the data are cross-sectional and therefore do not contain information about the causal relationships between mental disorders, disclosure of homosexual behaviors, and other factors that may affect lifetime suicidal behaviors, and could not tell whether those who are engaging in suicidal ideation and/or planning will go on to make a suicide attempt. Thus, a longitudinal study would provide an important complement to this study. Fourth, although RDS has been used widely to estimate the characteristics of hard-to-reach populations, the utility of RDS in mental health survey remains largely unknown; caution is required when interpreting the results. Finally, we selected the 807 MSM aged 18–65 from four cities in northeastern China, so our findings may not be generalizable to other regions of China.

## Conclusions

The results of this study provide clear evidence that Chinese MSM have increased risk for suicidal behaviors. Because of the potentially severe consequences of suicidal behaviors and the high prevalence of risk factors for suicidal behaviors in the estimated 18 million Chinese MSM, more efforts are needed to increase the tolerance and reduce the stigmatization and discrimination directed toward homosexuals in Chinese society. Early detection and effective treatment of psychiatric disorders and their comorbidities is crucial for reducing the suicide rates in Chinese MSM.

### Ethics and consent to participant

The study was conducted in accordance with the Declaration of Helsinki on ethical principles for medical research involving human subjects. The ethics committee of Liaoning Provincial Center for Disease Control and Prevention (LNCDCP) approved the study. All subjects gave written informed consent after the study objectives were explained, and all subjects were free to withdraw at any time without giving any reason.

### Consent to publish

Not applicable in this section.

### Availability of data and materials

The data will not be shared because of our agreement with the MSM subject.

## Abbreviations

MSM: men who have sex with men
OR: odds ratios
WMH-CIDI: World Mental Health Composite International Diagnostic Interview
RDS: respondent-driven sampling
LNCDCP: Liaoning Provincial Center for Disease Control and Prevention
CIDI 1.0: Composite International Diagnostic Interview Version 1.0
GAD: generalized anxiety disorder
OCD: obsessive compulsive disorder
SMM: sexual minority men
GRSEs: gay related stressful events
RRs: relative risks
